# Hospital Needs

**Published:** 1912-11-02

**Authors:** 


### Hospital Needs.

### HANDY URINE TESTS.

We have received from the Endolytic Tube Company, Hampton-on-Thames, samples of their Endolytic Tubes for clinical diagnosis. These tubes are thoroughly deserving of a trial by every practitioner, for they are clean, reliable, efficient, and rapid. The principle on which the test is applied is capillary attraction : a thread-bore tube filled with the reagent is opened at both ends—the file notch rendering it easy to snap off the thin end of the tube—is placed in a drop of urine to be tested, which is taken from the chamber vessel and placed on a piece of ordinary glazed notepaper. The urine runs up into the tube, mingles with the testing solution, and, if the suspected abnormal constituent is present, the reaction at once takes place. We have tested three solutions—that for albumen (salicyl sulphonic acid), which is admirable for the detection of both albumen and albumose; that for glucose, which is the ordinary Fehling's test (the tube being gently heated over a match flame); and that for diacetic acid, which is the well-known ferric chloride test. A fourth test—the sodium nitro-prusside test for acetone—is also supplied in these tubes. The Endolytic Tubes make urine testing an easy matter for the busiest practitioner; their cleanliness and convenience should appeal to every medical man, while their simplicity and compactness render them excellent addenda to the physician's pocket-case. The Company supplies them in a neat nickle pocket-case, which carries 100 tubes, at a price of 5s.

### THE FLOOR PROBLEM.

Secondary in importance to the material used for the floors of corridors, theatres, and wards, whether teak, terrazzo, mosaic, or tiles, is the method of washing or polishing them. "Gospo," a cleansing powder, manufactured by Gospo, Limited, 33 Waterloo Road, London, S.E., is a substitute for soft soap. It is recommended as being free from animal fats, and therefore leaves the floors bright without being slippery. Everyone knows how ordinary soap makes the floor look bright whilst it is being washed only to leave it dull when dry, and if for no other reason should make a personal practical test of "Gospo." Its uses are, however, not confined to floor polishing; general cleaning purposes, such, for instance, as the cleansing of baths, are among its useful applications.

### AN UNINTENTIONAL ADVERTISEMENT.

A new panel, executed by Mr. Frank O. Salisbury, has recently been placed in the Royal Exchange—the second on the left as you enter from the west side. King Alfred is depicted directing the rebuilding of London. The King has halted to consult his architect, on whose gorgeously decorated gown gilt circles and semi-circles magically form the word "Oxo" three times. The representation is clearer when viewed from the side than from the front. This curious and unintentional Oxo advertisement must, we feel sure, be the first one of its kind which has ever appeared.

